# The association of low complement with disease activity in systemic sclerosis: a prospective cohort study

**DOI:** 10.1186/s13075-016-1147-2

**Published:** 2016-10-22

**Authors:** James Esposito, Zoe Brown, Wendy Stevens, Joanne Sahhar, Candice Rabusa, Jane Zochling, Janet Roddy, Jennifer Walker, Susanna M. Proudman, Mandana Nikpour

**Affiliations:** 1 Department of Medicine, The University of Melbourne at St Vincent’s Hospital (Melbourne), 41 Victoria Parade, Fitzroy, VIC 3065 Australia; 2 Department of Rheumatology, St Vincent’s Hospital (Melbourne), 41 Victoria Parade, Fitzroy, VIC 3065 Australia; 3 Department of Rheumatology, Monash Health and Monash University, 246 Clayton Road, Clayton, VIC 3168 Australia; 4 Department of Medicine, Monash Health and Monash University, 246 Clayton Road, Clayton, VIC 3168 Australia; 5 Department of Rheumatology, Menzies Institute for Medical Research, Private Bag 23, Hobart, TAS 7001 Australia; 6 Department of Rheumatology, Royal Perth Hospital, 197 Wellington Street, GPO Box X2213, Perth, WA 6001 Australia; 7 Department of Rheumatology, Flinders Medical Centre, Flinders Drive, Bedford Park, SA 5042 Australia; 8 Rheumatology Unit, Royal Adelaide Hospital, North Terrace, Adelaide, SA 5000 Australia; 9 Discipline of Medicine, University of Adelaide, Adelaide, SA 5000 Australia

**Keywords:** Systemic sclerosis, Complement, Disease activity

## Abstract

**Background:**

In some rheumatic diseases such as systemic lupus erythematosus (SLE), low serum complement (‘hypocomplementaemia’) is a feature of active disease. However, the role of hypocomplementaemia in systemic sclerosis (SSc) is unknown. We sought to determine the frequency, clinical associations and relationship to disease activity of hypocomplementaemia in SSc.

**Methods:**

The study included 1140 patients fulfilling the 2013 American College of Rheumatology criteria for SSc. Demographic, serological and clinical data, obtained prospectively through annual review, were analysed using univariable methods. Linear and logistic regression, together with generalised estimating equations, were used to determine the independent correlates of hypocomplementaemia ever, and at each visit, respectively.

**Results:**

At least one episode of hypocomplementaemia (low C3 and/or low C4) occurred in 24.1 % of patients over 1893 visits; these patients were more likely to be seropositive for anti-ribonucleoprotein (OR = 3.8, *p* = 0.002), anti-Ro (OR = 2.2, *p* = 0.002), anti-Smith (OR = 6.3, *p* = 0.035) and anti-phospholipid antibodies (OR = 1.4, *p* = 0.021) and were more likely to display features of overlap connective tissue disease, in particular polymyositis (OR = 16.0, *p* = 0.012). However, no association was found between hypocomplementaemia and either the European Scleroderma Study Group disease activity score or any of its component variables (including erythrocyte sedimentation rate) in univariate analysis. Among patients with SSc overlap disease features, those who were hypocomplementaemic were more likely to have digital ulcers (OR = 1.6, *p* = 0.034), tendon friction rubs (OR = 2.4, *p* = 0.037), forced vital capacity <80 % predicted (OR = 2.9, *p* = 0.008) and lower body mass index (BMI) (OR for BMI = 0.9, *p* < 0.0005) at that visit, all of which are features associated with SSc disease activity and/or severity.

**Conclusions:**

While hypocomplementaemia is not associated with disease activity in patients with non-overlap SSc, it is associated with some features of increased SSc disease activity in patients with overlap disease features.

## Background

Systemic sclerosis (SSc) or scleroderma is a systemic autoimmune disorder of unknown aetiology associated with substantial morbidity and mortality [1]. The hallmark pathological features of SSc are vasculopathy, inflammation and progressive perivascular and interstitial fibrosis [2]. Prominent clinical manifestations of SSc include skin thickening, Raynaud’s phenomenon, digital ulcers, gut involvement (gastro-oesophageal reflux disease, intestinal hypomotility and pseudo-obstruction), pulmonary arterial hypertension (PAH), interstitial lung disease (ILD) and renal crisis [3]. An ‘SSc overlap’ syndrome is considered present when a patient with SSc also has clinical and/or serological evidence of one or more of systemic lupus erythematosus (SLE), polymyositis, Sjögren’s syndrome or rheumatoid arthritis [2].

As in other systemic autoimmune diseases, disease activity leads to irreversible organ damage. Activity reflects features of the disease process that vary over time and are potentially reversible with intervention or spontaneously. Quantifying active disease would assist clinicians in assessment and management of disease activity, which might in turn prevent damage and improve outcomes [4]. Assessing disease activity is necessary for staging the disease and predicting prognosis. It is also useful for distinguishing between those patients requiring aggressive treatment and those for whom symptomatic treatment may be sufficient, as well as for monitoring response to treatment.

In certain rheumatic diseases characterised by distinct episodes of inflammation, such as SLE, validated measures already exist to assist clinicians in the assessment of disease activity [5]. There have been several endeavours to develop disease activity criteria for SSc. The European Scleroderma Study Group (EScSG) activity index was constructed on the basis of evaluation of features of a large multicentre cohort of patients with SSc, compared with a gold standard of disease activity assessment by three experts [6–8]. Univariate analysis of symptoms, signs and laboratory tests was performed to select single items that were significantly associated with the consensus disease activity score. The final index comprised ten components, including clinical features such as arthritis, modified Rodnan skin score (MRSS) and digital ulcers; patient-reported changes in cardiopulmonary, skin and vascular symptoms; and erythrocyte sedimentation rate (ESR) [6, 7]. Hypocomplementaemia was also included [7, 8], but it is not yet known whether low complement is truly a marker of disease activity in SSc.

Various candidate serological markers have been proposed to be related to disease activity in SSc, such as soluble interleukin-2 receptor (sIL-2R), type III procollagen aminopeptide (PIIINP) and von Willebrand factor propeptide [9–11]. However, in patients with SSc, changes in these markers have not been reflected in clinical responses to therapy. For example, chlorambucil administration was followed by normalization of sIL-2R levels but had no effect on disease activity [12]. Similarly, α-interferon administration resulted in a decrease in PIIINP levels without any improvement in disease activity assessed using a validated skin score, grip strength, measurements of digital contractures, Ritchie index, assessment of muscle weakness, and tendon friction rubs [13].

Low serum complement is a candidate serological marker of disease activity in SSc. Aberrant activation of the complement system is implicated in the pathogenesis of a number of systemic autoimmune disorders [14]. For example, in SLE, immune complex formation triggers the complement cascade via the classical pathway, and the resulting low concentrations of complement components C3 and C4 are found in many patients with active and severe SLE [15, 16]. It has been shown that the manifestations of SSc are due to derangements in both innate and adaptive immunity. The association of Toll-like receptor (TLR) signalling variations with SSc suggests that TLR pathways, and hence complement activation and consumption, may play a role in the pathogenesis of SSc [17]. However, unlike SLE, wherein low serum complement is a validated measure of disease activity, the relationship between hypocomplementaemia and disease activity in SSc is less clear.

The aim of this study was to evaluate the frequency of hypocomplementaemia in a cohort of patients with SSc and to evaluate which clinical and serological features are significantly associated with hypocomplementaemia. We hypothesised that hypocomplementaemia is associated with clinical features of disease activity such as arthritis, tendon friction rubs, low body mass index (BMI) (a marker of severe SSc gastrointestinal involvement) and low forced vital capacity (FVC) (a measure of severe ILD).

## Methods

### Patients

Patients were recruited from the Australian Scleroderma Cohort Study, a prospective multi-centre cohort study of risk factors for clinically important outcomes in SSc. All patients fulfilled the 2013 American College of Rheumatology classification criteria for SSc [18]. Patients were recruited from multiple Australian centres that specialize in the care of patients with SSc, which included St Vincent’s Hospital and Monash Medical Centre, Victoria; Royal Prince Alfred and St George Hospitals, New South Wales; Sunshine Coast Rheumatology and Prince Charles Hospital, Queensland; Royal Adelaide Hospital, The Queen Elizabeth Hospital and Flinders Medical Centre, South Australia; Royal Perth Hospital, Western Australia; and Menzies Institute for Medical Research, Tasmania. The study was approved by the human research ethics committees of each of the participating centres.

Consent was obtained from all patients prior to the collection of demographic and disease-related data (including clinical and laboratory data) according to a standardized protocol at recruitment and at each subsequent annual review. Patients were included if they had at least one annual visit where complement levels had been recorded, along with demographic, clinical and laboratory data (see below).

### Measurement of complement levels

C3 and C4 levels were measured prospectively at each annual visit in the laboratories of each of the recruiting centres using sera obtained at recruitment and at each subsequent review by performing nephelometry. Complement levels were defined as being either normal or low according to local laboratory standards. Patient visits were then recorded as normocomplementaemic if the patient had a normal C3 and C4 result or hypocomplementaemic if the patient had a low C3 and/or C4.

### Demographic, clinical and laboratory variables

All data were collected prospectively.

#### Data collected at recruitment

Demographic data collected at recruitment included age at disease onset and age at recruitment. Disease onset was defined according to the age at which the first non-Raynaud’s phenomenon manifestation of SSc occurred. Sex and race were also recorded; race was categorized as white, Asian, Aboriginal or Torres Strait Islander, or other. Disease duration was defined as the time elapsed from disease onset to recruitment, and length of follow-up was defined as the time elapsed from recruitment until the date at which the data were censored for analysis.

Disease-related data gathered included SSc subtype (diffuse or limited) defined according to the LeRoy criteria [19]. The autoantibody status of each patient was determined at recruitment using immunofluorescence for antinuclear antibodies and commercially available assays for antibodies to extractable nuclear antigens Scl-70, Jo-1, ribonucleoprotein (RNP), Ro, La, Smith (Sm), and polymyositis scleroderma (PM-Scl) (enzyme-linked immunosorbent assay [ELISA]; ORGENTEC Diagnostika, Mainz, Germany); antibodies to double-stranded DNA (anti-dsDNA) (Amerlex radioimmunoassay; Trinity Biotech, Bray, Ireland); antibodies to RNA polymerase III (QUANTA Lite RNA Pol III; Inova Diagnostics, San Diego, CA, USA; and MBL Anti-RNA Pol III; MBL International, Woburn, MA, USA); anti-neutrophil cytoplasmic antibodies (ANCA), including proteinase-3 or myeloperoxidase specificity (ELISA; ORGENTEC Diagnostika); rheumatoid factor and anti-phospholipid antibodies, including anti-cardiolipin antibodies (Vital Diagnostics, Bella Vista, Australia); and anti-β_2_-glycoprotein antibodies (ELISA; ORGENTEC Diagnostika).

#### Data collected at each annual visit

Disease manifestations were recorded at each visit. Raynaud’s phenomenon was defined on the basis of characteristic colour changes in the extremities. The presence of puffy digits (scleroderma), tendon friction rubs, synovitis and muscle atrophy was based on examination findings of the patient’s rheumatologist. The MRSS [20] was also calculated and recorded at each visit.

Persistent sicca symptoms were defined as dry eyes or dry mouth at two or more annual visits. Gastrointestinal symptoms, including reflux requiring treatment with proton pump inhibitor, dysphagia, post-prandial bloating, vomiting, diarrhoea (more than three motions per day), constipation (fewer than one motion per day) or anal incontinence (faecal soiling) not due to other causes, were also recorded. Gastric antral vascular ectasia (GAVE) and oesophageal stricture were defined on the basis of endoscopy. Bowel dysmotility was defined on the basis of barium and nuclear medicine studies, antibiotic response and characteristic symptoms. Pseudo-obstruction was defined as the presence of clinical features suggestive of intestinal obstruction in the absence of an anatomical lesion.

Myocardial disease was defined on the basis of endomyocardial biopsy or as the presence of conduction defects, arrhythmias, and right ventricular or left ventricular dysfunction on echocardiography in the absence of other causes. Pericardial effusions were defined on the basis of an echocardiogram showing other than a small (<1-cm thickness), non-significant pericardial effusion.

PAH was defined on the basis of right heart catheterization as a mean pulmonary arterial pressure ≥25 mmHg and a pulmonary arterial wedge pressure ≤15 mmHg. The 6-minute walk distance was also recorded each year as a measure of PAH severity.

ILD was defined on the basis of characteristic changes visualised by chest high-resolution computed tomography. Pulmonary function tests were performed at each annual visit, and forced vital capacity (FVC) and diffusing capacity of the lung for carbon monoxide (DLCO) were recorded as percent predicted values, with FVC used as a measure of ILD activity and/or severity.

Renal crisis was defined as any two of the following three criteria: new-onset severe hypertension (≥180 mmHg systolic and/or ≥100 mmHg diastolic) without an alternate aetiology, microangiopathic haemolytic anaemia, or rising creatinine.

SSc treatment was recorded at each visit as follows: corticosteroids (oral prednisolone), immunosuppressives (leflunomide, methotrexate, azathioprine, penicillamine, hydroxychloroquine, mycophenolate, cyclophosphamide and calcineurin inhibitors), biologic therapies (tumour necrosis factor-α inhibitors, tocilizumab, abatacept, anti-CD20 antibodies and other B-cell modulators) and home oxygen.

### Definition of SSc overlap syndrome

The presence of ‘overlap disease’ was recorded at each annual visit and was defined as evidence of SSc together with characteristic symptoms or signs of overlap disease, which included persistent sicca symptoms, episodes of inflammatory myositis (muscle weakness together with two or more of elevated serum creatinine kinase, characteristic changes on electromyography and/or magnetic resonance imaging, or muscle biopsy showing typical histopathological changes), or a rheumatologist’s report of overlap with another rheumatic disease, such as rheumatoid arthritis, polymyositis, dermatomyositis, SLE or Sjögren’s syndrome.

### European Scleroderma Study Group disease activity score

The EScSG disease activity score and the presence of its ten components were recorded at each visit: scleroderma; digital necrosis; arthritis; MRSS >14; DLCO <80 % predicted; ESR >30 mm/h; hypocomplementaemia (low C3 and/or C4); and patient-reported changes in cardiopulmonary, vascular or skin symptoms in the preceding month. One point is assigned for the presence of each feature, and scores can range from 0 to 10. However, because hypocomplementaemia is one of the ten variables in the EScSG activity score, we calculated a score based on the other nine variables only, in order to avoid over-correlation in subsequent analysis. Therefore, final scores ranged from 0 to 9, with higher scores indicating higher disease activity.

### Physician global assessment

Physician global assessments of overall health, disease activity and disease damage were also recorded prospectively at each annual visit using a visual analogue scale. Physician global assessments entail the physician’s taking note of all the available clinical and laboratory information in order to assign a score that ranges between 0 and 10 for each of overall health, disease activity and disease-related damage. Higher scores represent worse overall health, higher disease activity or greater disease-related damage.

### Statistical analysis

Descriptive statistics (mean ± SD, median [interquartile range], minimum and maximum, and number [percent]) were used to describe the characteristics of the patients and the data set. As there was <5 % missing data for any particular variable, we did not impute missing data.

Univariable comparisons between patients who were persistently normocomplementaemic during follow-up and patients who had experienced at least one episode of hypocomplementaemia during the course of the study were made using the *t* test for continuous variables, the chi-square test for categorical variables and the Wilcoxon signed-rank test for ordinal variables. In this analysis, disease manifestations were defined as ‘present ever’. Additional analyses were performed comparing the two groups described above (persistent normocomplementaemia and at least one episode of hypocomplementaemia) with those who had persistent hypocomplementaemia at every visit during follow-up.

In multivariable analyses, the association between disease features at each visit and hypocomplementaemia at that particular visit was analysed. Here, we used generalised estimating equations (GEEs) to account for the expected correlation that arises when repeated measurements are taken from the same individual at multiple visits over time [21]. Another advantage of this method of analysis is that it overcomes any potential misclassification error that might arise from dividing patients into subgroups based on arbitrary definitions such as hypocomplementaemia ever, persistent hypocomplementaemia and persistent normocomplementaemia. Furthermore, as there is currently no consensus regarding meaningful changes in clinical parameters from one visit to the next, the use of GEEs enables evaluation of the relationship between hypocomplementaemia at a particular visit and actual clinical parameters at that particular visit.

**Fig. 1:**
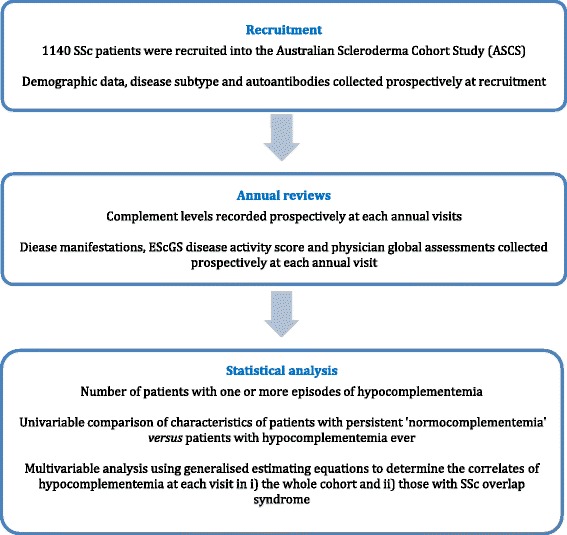
Study design. *EScSG* European Scleroderma Study Group, *SSc* Systemic sclerosis

All statistical analyses were performed using STATA 14 software (StataCorp, College Station, TX, USA). All *p* values were two-tailed, and statistical significance was defined as *p* ≤ 0.05. The overall study design and planned analyses are presented in Fig. 1.

## Results

This study included 1140 patients with SSc who had complement levels recorded prospectively at one or more visits.

### Characteristics of cohort and data set

The cohort presented in this study is summarized in Tables 1 and 2. Among the 1140 patients included in this study, the average age of patients was 46.0 ± 14.1 years at SSc diagnosis and 57.4 ± 12.3 years at recruitment, with a mean follow-up time of 3.4 ± 1.7 years. In terms of demographics, 87.2 % of patients were female, 93.3 % were white, 4.7 % were Asian, 1.3 % were Australian Aboriginal or Torres Strait Islander and 0.9 % were of other racial origin. The mean disease duration at recruitment was 11.3 ± 9.9 years, with 14.3 % of patients having a disease duration ≤2 years and 31.2 % of patients having a disease duration ≤5 years at recruitment. Diffuse SSc was present in 27.2 % of patients, and 72.8 % of patients had limited SSc.

**Table 1:** Patient characteristics (*n* = 1140)

Characteristic	*n* (%) or mean ± SD
Age at diagnosis, years	46.0 ± 14.1
Age at recruitment, years	57.4 ± 12.3
Follow-up duration, years	3.4 ± 1.7
Sex	
Male	148 (13.0 %)
Female	992 (87.2 %)
Race	
White	1012 (93.3 %)
Asian	50 (4.7 %)
Australian Aboriginal or Torres Strait Islander	14 (1.3 %)
Other	9 (0.9 %)
Disease duration at recruitment^a^	11.3 ± 9.9
≤ 2 years	163 (14.3 %)
≤ 5 years	356 (31.2 %)
Disease subtype^b^	
Diffuse	310 (27.2 %)
Limited	830 (72.8 %)
Overlap disease features present ever^c^	267 (23.4 %)
Rheumatoid arthritis	22 (1.9 %)
Polymyositis	6 (0.5 %)
Dermatomyositis	1 (0.01 %)
Sjögren’s syndrome	21 (1.8 %)
SLE	9 (0.8 %)
Serological profile at recruitment	
Anti-centromere ANA	517 (46.2 %)
Anti-Scl-70 antibodies	167 (15.2 %)
Anti-RNAP antibodies	84 (13.3 %)
Anti-U1 RNP antibodies	22 (2.0 %)
Anti-Ro antibodies	72 (6.6 %)
Anti-La antibodies	18 (1.6 %)
Anti-Sm antibodies	6 (0.6 %)
Anti-PM-Scl antibodies	15 (1.4 %)
Anti-dsDNA antibodies	26 (3.1 %)
Anti-Jo-1 antibodies	5 (0.5 %)
ANCA	134 (12.9 %)
MPO specificity	17 (1.6 %)
PR-3 specificity	22 (2.1 %)
Rheumatoid factor	288 (27.2 %)
Anti-phospholipid antibodies	272 (25.7 %)
Cardiolipin IgM	165 (65.0 %)
Cardiolipin IgG	105 (39.3 %)
Anti-β_2_-glycoprotein antibody	84 (33.0 %)
Lupus anticoagulant	26 (3.1 %)

*Abbreviations: ANA* Anti-nuclear antibody, *Anti-Scl-70* Anti-scleroderma-70 antibodies, *Anti-U1 RNP* Anti-ribonucleoprotein antibodies, *Anti-Sm* Anti-Smith antibodies, *Anti-PM-Scl* Anti-polymyositis scleroderma antibodies, *Anti-dsDNA* Anti-double-stranded DNA antibodies, *Anti-RNAP* Anti-RNA polymerase antibodies, *ANCA* Anti-neutrophil cytoplasmic antibodies, *MPO* Myeloperoxidase, *PR-3* Proteinase-3

^a^Since onset of first non-Raynaud’s phenomenon disease manifestation

^b^Disease subtype based on extent of skin involvement, with limited disease being confined to the extremities distal to elbows and knees, as well as the face

^c^Actual overlap disease features specified for only a proportion of patients classified by the treating physician as having ‘SSc overlap syndrome’

**Table 2:** Disease manifestations in cohort (*n* = 1140)

Characteristics	*n* (%) or median (25th–75th IQR)
Disease manifestation^a^	
Raynaud’s phenomenon	1065 (94.0 %)
Digital ulcers	345 (30.5 %)
Digital gangrene/amputation	93 (8.2 %)
Telangiectasia	947 (83.8 %)
Calcinosis	439 (38.9 %)
Scleroderma	763 (67.8 %)
Tendon friction rub	112 (10.0 %)
Joint contracture	431 (38.3 %)
Synovitis	323 (28.7 %)
Muscle atrophy	210 (18.7 %)
MRSS score >20	207 (18.5 %)
Myocardial disease	87 (7.6 %)
Pericardial effusion^b^	70 (6.2 %)
PAH^c^	298 (26.9 %)
ILD^d^	340 (30.1 %)
Gastrointestinal involvement^e^	638 (56.3 %)
GAVE	113 (10.0 %)
Reflux oesophagitis	958 (84.3 %)
Oesophageal stricture	207 (18.3 %)
Oesophageal dysmotility	457 (40.6 %)
Bowel dysmotility	297 (26.3 %)
Pseudo-obstruction	37 (3.3 %)
Renal crises^f^	44 (3.9 %)
eGFR <60 ml/minute	297 (26.4 %)
Myositis^g^	58 (6.0 %)
CRP >8 mg/L	333 (29.7 %)
ESR >30 mm/h	323 (28.8 %)
Blood CK >200 IU/L	122 (11.0 %)
Anaemia^h^	403 (35.5 %)
FVC <80 % predicted	298 (26.9 %)
DLCO <80 % predicted	832 (79.9 %)
EScSG disease activity score^i^	2.5 (1–4)
Treatments^a^	
Corticosteroids	506 (44.4 %)
Immunotherapy^j^	495 (43.4 %)
Biological therapy^k^	16 (1.4 %)
Home oxygen	41 (3.6 %)
Physician global assessments^*l*^	
Overall health^m^	4 (3–6)
Activity^n^	3 (2–5)
Damage^n^	4 (3–6)

*Abbreviations: MRSS* Modified Rodnan skin score, *EScSG* European Scleroderma Study Group, *PAH* Pulmonary arterial hypertension, *ILD* Interstitial lung disease, *GAVE* Gastric antral vascular ectasia, *eGFR* Estimated glomerular filtration rate, *CRP* C-reactive protein, *ESR* Erythrocyte sedimentation rate, *CK* Creatinine kinase, *FVC* Forced vital capacity, *DLCO* Diffusing capacity of the lung for carbon monoxide corrected for haemoglobin

^a^Ever from disease onset to most recent visit

^b^Pericardial effusion defined by echocardiography

^c^PAH defined by right heart catheterization with a mean pulmonary artery pressure ≥25 mmHg and a pulmonary arterial wedge pressure ≤15 mmHg

^d^Pulmonary fibrosis defined by chest high-resolution computed tomography

^e^Gastrointestinal symptoms defined as the presence of any of reflux, dysphagia, post-prandial bloating, vomiting, constipation, diarrhoea or anal incontinence as defined in text

^f^Renal crisis defined as the presence of at least two of the following: new-onset hypertension, rising creatinine or microangiopathic haemolytic anaemia

^g^Myositis defined as either definite (biopsy), suspected (CK or electromyogram) or possible (magnetic resonance imaging scan)

^h^Anaemia defined as haemoglobin level <135 g/L in males and <120 g/L in females

^i^Calculated without hypocomplementaemia. Final scores range from 0 to 9, with higher scores indicating higher disease activity

^j^Includes leflunomide, methotrexate, azathioprine, penicillamine, hydroxychloroquine, mycophenolate, cyclophosphamide and calcineurin inhibitors

^k^Includes tumour necrosis factor alpha inhibitors, tocilizumab, abatacept, anti-CD20 antibodies and other B-cell modulators

^*l*^Highest score ever recorded over the study period

^m^Scores range from 0 to 10, with higher scores being indicative of worse overall health

^n^Scores range from 0 to 10, with higher scores being indicative of higher disease activity or damage

SSc overlap disease features were present in 23.4 % of patients. The autoantibody profile of patients is summarized in Table 1. Autoantibodies that were found in a significant proportion of the cohort included anti-Scl-70 (15.2 %), anti-centromere (46.2 %), anti-RNA polymerase (13.3 %), anti-dsDNA antibodies (13.3 %), ANCA (12.9 %), rheumatoid factor (27.2 %), anti-phospholipid (25.7 %) and anti-β_2_-glycoprotein (33.0 %) antibodies.

Characteristics of the data set pertaining to complement measurements are summarized in Table 3. Hypocomplementaemia was present at recruitment in 13.2 % of patients and thereafter present ever from recruitment to most recent visit in 24.1 % of patients. The median number of complement measurements per patient was 2, with an interquartile range of 1–3. The minimum number of measurements was 1, and the maximum number of measurements was 8. The average time interval between measurements was 1.2 ± 0.4 years. A total of 1893 individual visit measurements of complement with corresponding clinical and serological data were available for analysis.

**Table 3:** Characterisation of complement levels (*n* = 1140)

Parameter	*n* (%) or mean ± SD or median (25th–75th IQR)
Hypocomplementaemia^a^	
At recruitment	150 (13.2 %)
Ever^b^	275 (24.1 %)
Number of complement measurements per patient	2 (1–3)
1	400 (35.1 %)
2	265 (23.3 %)
3	196 (17.2 %)
4	129 (11.3 %)
5	85 (7.5 %)
6	50 (4.4 %)
7	14 (1.2 %)
8	1 (0.1 %)
Total number of complement measurements in data set	1893
Time interval between complement measurements, years	1.2 ± 0.4

^a^A low C3 and/or C4 result

^b^At least one episode of hypocomplementaemia over the study period

### Univariable analyses

Univariable comparisons between patients who had persistent normocomplementaemia during follow-up and patients who had experienced at least one episode of hypocomplementaemia during the course of the study are summarized in Tables 4 and 5. Patients who had ever been hypocomplementaemic were more likely to have features of overlap disease present (29.5 % vs. 21.5 %, *p* = 0.007), in particular polymyositis (1.8 % vs. 0.1 %, *p* = 0.001). Hypocomplementaemia was also found to be associated with a number of autoantibodies typically associated with other rheumatic diseases, which included anti-RNP (4.5 % vs. 1.2 %, *p* = 0.001), anti-Ro (10.8 % vs. 5.2 %, *p* = 0.001), anti-Sm (1.5 % vs. 0.2 %, *p* = 0.016) and anti-phospholipid (31.3 % vs. 24.0 %, *p* = 0.02) antibodies. Patients with hypocomplementaemia were also more likely to have a BMI <20 kg/m^2^ (20.5 % vs. 13.0 %, *p* = 0.003), a feature of more active and severe gastrointestinal involvement; scleroderma (72.8 % vs. 66.2 %, *p* = 0.044); and muscle atrophy (23.2 % vs 17.2 %, *p* = 0.029). A significant association with pericardial effusions (9.1 % vs. 5.3 %, *p* = 0.023) was also found.

**Table 4:** Univariable comparison of demographics, disease subtypes and serological profiles in patients with persistent normocomplementaemia and those with at least one episode of hypocomplementaemia

Characteristic	Persistent normocomplementaemia (*n* = 865)	At least one episode of hypocomplementaemia (*n* = 275)	
	*n* (%) or mean ± SD or median (IQR)	*n* (%) or mean ± SD or median (IQR)	*p* Value
Age at diagnosis, years	46.4 ± 0.5	44.6 ± 0.8	0.071
Sex			
Male	113 (13.1 %)	35 (12.7 %)	0.89
Female	752 (86.9 %)	240 (87.3 %)	0.89
Disease duration	11.3 ± 0.4	11.6 ± 0.7	0.71
≤ 2 years	117 (14.5 %)	46 (17.5 %)	0.24
≤ 5 years	268 (33.2 %)	88 (33.5 %)	0.94
Disease type			
Diffuse	238 (27.5 %)	72 (26.2 %)	0.67
Limited	627 (72.5 %)	203 (73.8 %)	0.67
Overlap features present^a^	186 (21.5 %)	81 (29.5 %)	0.007
Rheumatoid arthritis	15 (1.7 %)	7 (2.6 %)	0.39
Polymyositis	1 (0.1 %)	5 (1.8 %)	0.001
Dermatomyositis	0 (0 %)	1 (0.4 %)	0.076
Sjögren’s syndrome	14 (1.6 %)	7 (2.6 %)	0.32
SLE	6 (0.7 %)	3 (1.1 %)	0.52
Serological profile^a^			
Anti-centromere ANA	391 (46.0 %)	126 (47.0 %)	0.084
Anti-Scl-70 antibodies	134 (16.1 %)	33 (12.3 %)	0.13
Anti-Jo-1 antibodies	4 (0.5 %)	1 (0.4 %)	0.82
Anti-RNP antibodies	10 (1.2 %)	12 (4.5 %)	0.001
Anti-Ro antibodies	43 (5.2 %)	29 (10.8 %)	0.001
Anti-La antibodies	12 (1.5 %)	6 (2.2 %)	0.38
Anti-Sm antibodies	2 (0.2 %)	4 (1.5 %)	0.016
Anti-PM-Scl antibodies	10 (1.2 %)	5 (1.9 %)	0.43
Anti-dsDNA antibodies	16 (2.5 %)	10 (4.9 %)	0.089
Anti-RNAP antibodies	61 (13.6 %)	23 (12.6 %)	0.74
ANCA	95 (12.1 %)	39 (15.2 %)	0.20
MPO specificity	14 (1.8 %)	3 (1.2 %)	0.50
PR-3 specificity	14 (1.8 %)	8 (3.1 %)	0.20
Rheumatoid factor	208 (26.1 %)	80 (31.3 %)	0.11
Anti-phospholipid antibodies	192 (24.0 %)	80 (31.3 %)	0.02
Cardiolipin IgM	115 (63.2 %)	50 (69.4 %)	0.351
Cardiolipin IgG	78 (41.5 %)	27 (34.2 %)	0.26
β_2_-glycoprotein	59 (32.8 %)	25 (33.3 %)	0.93
Lupus anti-coagulant	15 (2.4 %)	11 (5.0 %)	0.056

*Abbreviations: ANA* Anti-nuclear antibody, *Anti-Scl-70* Anti-scleroderma-70 antibodies, *Anti-RNP* Anti-ribonucleoprotein antibodies, *Anti-Sm* Anti-Smith antibodies, *Anti-PM-Scl* Anti-polymyositis scleroderma antibodies, *Anti-dsDNA* Anti-double-stranded DNA antibodies, *Anti-RNAP* Anti-RNA polymerase antibodies, *ANCA* Anti-neutrophil cytoplasmic antibodies, *MPO* Myeloperoxidase, *PR-3* Proteinase-3, *Ig* Immunoglobulin, *SLE* Systemic lupus erythematosus

^a^Ever from disease onset to most recent visit

**Table 5:** Univariable associations of hypocomplementaemia with clinical manifestations, treatment, European Scleroderma Study Group disease activity score and physician global assessments

Characteristic	Persistent normocomplementaemia (*n* = 859)	At least one episode of hypocomplementaemia (*n* = 275)	
	*n* (%) or mean ± SD or median (IQR)	*n* (%) or mean ± SD or median (IQR)	*p* Value
Disease manifestation^a^			
Raynaud’s phenomenon	803 (93.5 %)	262 (95.6 %)	0.19
BMI <20 kg/m^2^	104 (13.0 %)	54 (20.5 %)	0.0030
Digital ulcers	263 (30.7 %)	82 (30.0 %)	0.848
Digital gangrene/amputation	76 (8.8 %)	17 (6.2 %)	0.17
Telangiectasia	716 (83.6 %)	231 (84.6 %)	0.68
Calcinosis	340 (39.7 %)	99 (36.3 %)	0.31
Scleroderma	565 (66.2 %)	198 (72.8 %)	0.044
Tendon friction rub	88 (10.3 %)	24 (8.8 %)	0.47
Joint contracture	332 (38.9 %)	99 (36.4 %)	0.46
Synovitis	241 (28.3 %)	82 (30.0 %)	0.59
Muscle atrophy	147 (17.2 %)	63 (23.2 %)	0.029
MRSS >20	158 (18.7 %)	49 (18.0 %)	0.81
Myocardial disease	70 (8.1 %)	17 (6.2 %)	0.30
Pericardial effusion	45 (5.3 %)	25 (9.1 %)	0.023
PAH	90 (10.4 %)	30 (10.9 %)	0.82
Pulmonary fibrosis	263 (30.8 %)	77 (30.1 %)	0.40
Gastrointestinal involvement	493 (57.5 %)	145 (52.7 %)	0.17
GAVE	86 (10.1 %)	27 (9.9 %)	0.94
Reflux oesophagitis	722 (83.8 %)	236 (85.8 %)	0.41
Oesophageal stricture	109 (12.8 %)	23 (8.4 %)	0.051
Oesophageal dysmotility	346 (40.5 %)	111 (40.8 %)	0.92
Bowel dysmotility	226 (26.4 %)	71 (25.9 %)	0.88
Pseudo-obstruction	25 (2.9 %)	12 (4.4 %)	0.23
Renal crises	36 (4.2 %)	8 (2.9 %)	0.34
eGFR <60 ml/minute	229 (22.8 %)	68 (25.0 %)	0.55
Myositis	37 (5.2 %)	21 (8.5 %)	0.055
CRP >8 mg/L	266 (31.1 %)	67 (24.9 %)	0.051
ESR >30 mm/h	252 (29.6 %)	71 (26.1 %)	0.27
Blood CK >200 IU/L	105 (12.1 %)	44 (16.0 %)	0.098
Anaemia	302 (35.1 %)	101 (36.7 %)	0.62
FVC <80 %	225 (26.9 %)	73 (27.0 %)	0.97
DLCO <80 %	631 (80.5 %)	201 (78.2 %)	0.43
Treatment^a^			
Corticosteroids	380 (43.9 %)	126 (45.8 %)	0.58
Immunotherapy	364 (42.1 %)	131 (47.6 %)	0.11
Biologic therapy	9 (1.0 %)	7 (2.6 %)	0.065
Home oxygen	32 (3.7 %)	9 (3.3 %)	0.74
EScSG score^b^	2.5 (1.4)	2 (1–4)	0.16
Physician global assessments^b^			
Health	4 (3–6)	4 (3–6)	0.25
Activity	3 (2–5)	3 (2–5)	0.076
Damage	4 (2.5–6)	4 (3–6)	0.44

*Abbreviations: MRSS* Modified Rodnan skin score, *EScSG* European Scleroderma Study Group, *PAH* Pulmonary arterial hypertension, *ILD* Interstitial lung disease, *GAVE* Gastric antral vascular ectasia, *BMI* Body mass index, *eGFR* Estimated glomerular filtration rate, *CRP* C-reactive protein, *ESR* Erythrocyte sedimentation rate, *CK* Creatinine kinase, *FVC* Forced vital capacity, *DLCO* Diffusing capacity of the lung for carbon monoxide corrected for haemoglobin

^a^Ever from disease onset to most recent visit

^b^Mean score from all visits

There was no difference in the mean EScSG disease activity score or physician global assessment score for overall health, disease activity and damage across all visits in patients who were normocomplementaemic compared with those who were hypocomplementaemic. In additional analyses, we found no significant differences between those who had at least one episode of hypocomplementaemia and persistent hypocomplementaemia during follow-up, possibly due to very few patients in the latter group (data not shown).

### Multivariable regression analyses

In the analyses described above, variables were defined as being ever present from disease onset to most recent review. We performed a second series of analyses using GEEs with data gathered at each visit. This set comprised a total of 1893 visits.

We analysed data for up to six reviews per patient. We specified an exchangeable working correlation structure to account for the within-individual correlation and computed robust standard errors on the parametric estimates. We ran a univariable model of EScSG disease activity score (with the complement component removed to avoid over-correlation) and a multivariable model containing all of the individual disease activity variables listed in the EScSG disease activity index, with the exception of the complement item, and with some additional disease activity variables (C-reactive protein [CRP] >8 mg/L and BMI) included on the basis of univariable analyses (Table 6). Because scleroderma, muscle atrophy and pericardial effusion, which were statistically significant in univariable analysis, were not statistically significant in multivariable analysis, these variables were removed. Despite lack of significance in a simple univariable comparison, in a multivariable GEE model, FVC <80 % predicted was statistically significant and therefore included in the final model. We ran the multivariable models for (1) the whole cohort (*n* = 886; 1893 visits in total) as well as (2) patients classified as having SSc overlap (*n* = 221; 628 visits in total) and (3) those with non-SSc overlap (*n* = 665; 1265 visits in total) (Table 6).

**Table 6:** Multivariable associations of hypocomplementaemia with features of disease activity at each visit among the whole cohort and analysis subsets determined using generalised estimating equations

	Whole cohort (*n* = 886; 1893 visits)		SSc with features of overlap disease (*n* = 221;628 visits)		SSc without features of overlap disease (*n* = 665; 1265 visits)	
Parameter	Odds ratio (95 % CI)	*p* Value	Odds ratio (95 % CI)	*p* Value	Odds ratio (95 % CI)	*p* Value
Self-reported worsening of cardiopulmonary, vascular or skin symptoms^a^	1.05 (0.57–1.94)	0.87	1.24 (0.48–3.18)	0.66	1.03 (0.45–2.35)	0.94
Digital ulcers/necrosis^a^	1.15 (0.84–1.56)	0.38	1.62 (1.04–2.51)	0.034	0.82 (0.53–1.27)	0.37
Scleroderma^a^	0.97 (0.76–1.23)	0.78	1.08 (0.75–1.58)	0.67	0.87 (0.63–1.18)	0.37
Tendon friction rubs^a^	1.15 (0.64–2.08)	0.64	2.31 (1.05–5.10	0.037	0.54 (0.19–1.53)	0.24
Synovitis/arthritis^a^	0.95 (0.69–1.31)	0.76	1.18 (0.73–1.91)	0.50	0.80 (0.51–1.25)	0.32
Modified Rodnan skin score >14^a^	0.98 (0.96–0.99)	0.013	0.97 (0.95–1.01)	0.087	0.98 (0.96–1.01)	0.16
Erythrocyte sedimentation rate >30 mm/h^a^	0.98 (0.98–0.99)	0.022	0.99 (0.99–1.01)	0.97	0.99 (0.97–0.99)	0.026
DLCO <80 % predicted^a^	0.81 (0.61–1.09)	0.17	1.14 (0.66–1.96)	0.65	0.72 (0.51–1.03)	0.073
C-reactive protein >8 mg/L^a^	1.01 (0.99–1.01)	0.90	0.97 (0.93–1.01)	0.073	1.01 (0.99–1.01)	0.41
BMI^b^	0.91 (0.88–0.94)	<0.0005	0.90 (0.85–0.95)	<0.0005	0.91 (0.87–0.95)	<0.0005
FVC <80 % predicted^b^	1.24 (0.78–1.97)	0.37	2.90 (1.32–6.38)	0.0080	0.87 (0.47–1.61)	0.65

*Abbreviations: BMI* Body mass index, *FVC* forced vital capacity, *DLCO* Diffusing capacity of the lung for carbon monoxide corrected for haemoglobin

All variables listed in the table were included in the final multivariable generalised estimating equation model

^a^Components of European Scleroderma Study Group disease activity index

^b^Additional disease activity variables entered into multivariable regression model on the basis of significance in univariable analysis

In the multivariable analysis using the entire cohort, significant correlates of hypocomplementaemia at each visit included a lower BMI (OR for BMI = 0.90, 95 % CI 0.88–0.94, *p* < 0.0005), a lower MRSS (OR for MRSS = 0.98, 95 % CI 0.96–0.99, *p* = 0.013) and a lower ESR (OR for ESR = 0.99, 95 % CI 0.98–0.99, *p* = 0.022). In patients with SSc overlap disease features, significant correlates of hypocomplementaemia at each visit included digital ulcers (OR = 1.62, 95 % CI 1.04–2.51, *p* = 0.034), tendon friction rubs (OR = 2.31, 95 % CI 1.05–5.10, *p* = 0.037), an FVC <80 % predicted (OR = 2.90, 95 % CI 1.32–6.38, *p* = 0.008) and a lower BMI (OR for BMI = 0.90, 95 % CI 0.85–0.95, *p* < 0.0005), all variables that are associated with increased SSc disease activity and/or severity. In patients without overlap disease features, significant correlates of hypocomplementaemia at each visit included a lower BMI (OR for BMI = 0.91, 95 % CI 0.87–0.95, *p* < 0.0005) and a lower ESR (OR for ESR = 0.99, 95 % CI 0.97–0.99, *p* = 0.026).

## Discussion

In this longitudinal cohort study of 1140 patients with SSc, we found that low serum complement occurred at least once in 23.4 % of patients over the study period. We also found that these patients were more likely to have SSc overlap disease features than patients who were normocomplementaemic (29.5 % vs. 21.5 %, *p* = 0.007), in particular polymyositis. We have also demonstrated that hypocomplementaemia is not a measure of disease activity when applied across the entire cohort of patients with SSc. However, to our knowledge, this is the first study to demonstrate that hypocomplementaemia is a marker of the presence of certain SSc disease features measured at each visit among the subset of patients with overlap disease features. These disease features include digital ulcers, tendon friction rubs, low BMI and low FVC, all of which are clinical features of SSc disease activity or severity. This in turn suggests a role for measurement of C3 and C4 in assessment and monitoring of disease activity in patients with overlap SSc.

We found that the prevalence of hypocomplementaemia at recruitment was 13.2 %, which is consistent with previous smaller studies [22, 23]. The finding that hypocomplementaemia is associated with the presence of overlap disease features in SSc is also consistent with trends identified by Hudson et al. in a cohort of 321 Canadian patients with SSc [23]. This suggests that complement consumption plays a significant role in the etiopathogenesis of SSc overlap disease.

Several small studies have cited a role for abnormal complement activation in SSc. Senaldi et al*.* found that the levels of serum complement fragments Ba, C3d and C4d were higher in patients with diffuse SSc than in patients with limited SSc and that both subtypes had serum complement fragment levels higher than those of normal patients, suggesting that complement consumption occurs in SSc and may also be related to disease activity and/or severity [24]. Batal et al. undertook a study of prognostic factors in systemic sclerosis renal crisis (SRC) in which they retrospectively analysed renal biopsies. They found that peritubular capillary C4d deposition was higher in patients with SRC than in both hypertensive non-SRC and normotensive non-SRC control subjects and suggested a role for immune complex- and antibody-mediated injury in SRC [25]. Whilst in vivo activation of the complement system in SSc remains a possibility, the general consensus is that complement consumption does not play a significant role in the pathophysiology of SSc and that therefore the presence of hypocomplementaemia in SSc signifies the presence of another overlapping disease. This is consistent with the results of our study, which demonstrated a significant association between hypocomplementaemia and the presence of SSc overlap disease, in particular polymyositis. However, it is intriguing to note that even among patients with overlap disease, low serum complement was associated with disease features classically associated with SSc itself, such as digital ulcers and tendon friction rubs.

Hypocomplementaemia is one of the ten variables listed in the EScSG disease activity index [26, 27]. However, its inclusion has been a point of controversy. In the present study, we were unable to demonstrate a consistent association between hypocomplementaemia and either the EScSG disease activity score (calculated on the basis of nine non-complement variables to avoid over-correlation between independent variables) or any of its component variables in the univariable analysis. In addition, the associations found in the multivariable analysis in the subset of patients with SSc without overlap disease features (non-overlap SSc) suggest that, compared with normocomplementaemic patients, hypocomplementaemic patients have lower EScSG disease activity scores and ESR levels, which is the opposite of what one might expect. This lack of expected correlation with SSc disease activity measures suggests that hypocomplementaemia is not a measure of disease activity in the majority of patients with SSc, which is consistent with the conclusions drawn from previous studies [23]. Only one study suggested the contrary. Cuomo et al. reported the associations between hypocomplementaemia and clinical status in a cohort of 302 Italian patients with SSc and excluded patients with overlap disease features. They found that 16.5 % of their cohort had hypocomplementaemia and that hypocomplementaemia was associated with significantly increased disease activity, significantly increased severity of disease manifestations in the skin, cardiovascular and respiratory systems, and greater functional disability [22]. However, it is unclear whether the results of their study are generalizable to other SSc cohorts, because there was a very high prevalence of patients with anti-Scl-70 and anti-centromere ANA. The latter autoantibodies in particular have been suggested to play a role in complement activation [28].

The lack of association between hypocomplementaemia and other potential markers of disease activity in the EScSG disease activity index may also speak to the latter’s not being a very good measure of ‘disease activity’ per se. A trend toward a significant difference in the physician global assessment of disease activity between the persistently normocomplementaemic and the hypocomplementaemic groups (*p* = 0.076) supports this statement. However, it is also possible that each item in the EScSG disease activity index measures disease activity independently of all other items. Furthermore, it must be noted that functional abnormalities in complement in SSc may occur in the absence of hypocomplementaemia.

To our knowledge, this is the first study to use GEE methods to definitively show that hypocomplementaemia is associated with some features of disease activity in patients with SSc who have overlap disease features. We found that within this subset of patients with SSc, those who were hypocomplementaemic were more likely to manifest digital ulcers, tendon friction rubs, an FVC <80 % predicted and a lower BMI at each visit, all of which are well-established features of disease activity and severity in non-overlap SSc itself. No association was found with either CRP or ESR, possibly because these inflammatory markers generally are not considered to be good measures of disease activity in SSc. In particular, it has been shown that CRP levels are not elevated in most patients with SSc and that CRP levels regress as disease duration increases [29].

This study is not without limitations. As patients had varying lengths of follow-up, some had more annual complement measurements than others, making it possible that there was a low complement reading by chance alone on one or more occasions. However, the use of GEEs for statistical analysis overcomes this issue to a degree because it enables hypocomplementaemia at each visit to be associated with disease features at that particular visit, eliminating the need to divide patients into subgroups based on arbitrary definitions such as hypocomplementaemia ever, persistent hypocomplementaemia and persistent normocomplementaemia.

## Conclusions

In this study, we have shown that hypocomplementaemia in SSc is associated with the presence of SSc overlap features, in particular SSc-polymyositis overlap syndrome. We have also confirmed the results of previous studies by showing that hypocomplementaemia is not a measure of disease activity in non-overlap SSc cohorts. However, to our knowledge, this is the first study to show that hypocomplementaemia is associated with features of disease activity in SSc overlap disease and that, in these patients, the measurement of complement levels is potentially useful for monitoring disease activity and response to treatment.

## Abbreviations

ANA: Anti-nuclear antibodies
ANCA: Anti-neutrophil cytoplasmic antibodies
Anti-dsDNA: Anti-double-stranded DNA
Anti-PM-Scl: Anti-polymyositis scleroderma antibodies
Anti-RNAP: Anti-RNA polymerase antibodies
Anti-Scl-70: Anti-scleroderma-70 antibodies
Anti-Sm: Anti-Smith
Anti-U1 RNP: Anti-ribonucleoprotein antibodies
BMI: Body mass index
CK: Creatine kinase
CRP: C-reactive protein
DLCO: Diffusing capacity of the lung for carbon monoxide
eGFR: Estimated glomerular filtration rate
ELISA: Enzyme-linked immunosorbent assay
EScSG: European Scleroderma Study Group
ESR: Erythrocyte sedimentation rate
FVC: Forced vital capacity
GAVE: Gastric antral vascular ectasia
GEE: Generalised estimating equation
Ig: Immunoglobulin
ILD: Interstitial lung disease
MPO: Myeloperoxidase
MRSS: Modified Rodnan skin score
PAH: Pulmonary arterial hypertension
PIIINP: Type III procollagen aminopeptide
PR-3: Proteinase-3 specificity
RNA Pol: RNA polymerase
RNP: Ribonucleoprotein
sIL-2R: Soluble interleukin-2 receptor
SLE: Systemic lupus erythematosus
SRC: Systemic sclerosis renal crisis
SSc: Systemic sclerosis
TLR: Toll-like receptor
